# Ultrasound-Guided Intranodal Lipiodol Lymphangiography for the Assessment and Treatment of Chylous Leaks: A Retrospective Case Series from a Single Center in Switzerland and a Systematic Review of the Literature

**DOI:** 10.3390/jcm13216432

**Published:** 2024-10-27

**Authors:** Stephanie Nicole Schulz, Almir Miftaroski, Benoit Rouiller, Bernard Egger, Jon A. Lutz, Lucien Widmer

**Affiliations:** 1 Department of Reconstructive, Esthetic, and Plastic Surgery, Geneva University Hospital, Rue Gabrielle-Perret-Gentil 4, 1205 Genève, Switzerland; 2 Department of General Surgery, Geneva University Hospital, Rue Gabrielle-Perret-Gentil 4, 1205 Genève, Switzerland; almir.miftaroski@hcuge.ch; 3 Department of Thoracic Surgery, Fribourg Cantonal Hospital, Chem. des Pensionnats 2/6, 1752 Villars-sur-Glâne, Switzerland; benoit.rouiller@h-fr.ch (B.R.); jon.lutz@h-fr.ch (J.A.L.); 4 Department of General Surgery, Fribourg Cantonal Hospital, Chem. des Pensionnats 2/6, 1752 Villars-sur-Glâne, Switzerland; bernard.egger@h-fr.ch; 5 Department of Diagnostic and Interventional Radiology, Fribourg Cantonal Hospital, Chem. des Pensionnats 2/6, 1752 Villars-sur-Glâne, Switzerland

**Keywords:** Lipiodol lymphangiography, chylous leaks, post operative

## Abstract

**Background:** Lymphatic leaks are well-known complications of major thoracic or abdominal surgeries, which significantly heighten morbidity and mortality rates. While the existing literature provides insights into managing these post-operative leaks, with a step-up approach from conservative measures (CMs) to surgical intervention, there are no standardized treatment guidelines. The purpose of this paper is to offer a management algorithm of post-operative lymphatic leaks based on a systematic literature review (SLR) of the therapeutic effect of Lipiodol lymphangiography (LL), completed by a case series of five patients who underwent LL in our department. **Methods:** In this IRB-approved study, we conducted an SLR following the PRISMA guidelines, using a PICOS. A quality assessment was performed for each study. The case series consisted of consecutive patients who underwent LL for diagnostic and therapeutic purposes at our institution between September 2018 and December 2020. **Results:** A total of 39 observational studies were included in the SLR comprising 11 retrospective case reviews (Group 1), and 3 case series as well as 25 case reports (Group 2). In total, these studies report cases of 557 patients (51.52% presenting oncological diagnoses; 43.98% having benefited from lymphadenectomy). Lymphatic or chylous fistulas were the most encountered complication, followed by chylothorax. The median volume of Lipiodol injected during lymphography was 11.7 mL (range: 9.8–75 mL). Overall, LL was technically successful in 77.7% (366/471) of patients. The clinical success of all technically successful LLs was 80.6% (295/366). Time-to-leak resolution after lymphography varied between 1 and 31 days. The factors associated with treatment failure were a high leak output (>500 mL/day) and Lipiodol extravasation on post-LL imaging. Our case series consisted of five patients (mean age: 62 ± 9.24 years; 20% female; 100% oncological diagnoses; 60% having beneficiated from lymphadenectomy). Technical and clinical successes were 80% (4/5) and 75% (3/4), respectively. Time-to-leak resolution varied between 1 and 4 days. The volume and technique of LL was not different from that identified in the SLR. **Conclusions:** LL is a safe procedure with high technical and clinical success rates that could be proposed as both a diagnostic and therapeutic solution for patients with post-operative central lymphatic lesions.

## 1. Introduction

Lymphatic leaks are well-known complications of major thoracic or abdominal surgery. Lesions of the peripheral lymphatic vessels result in the accumulation of clear fluid, giving rise to conditions such as lymphoceles, lymphorrhea, lymphatic fistulas, or lymphatic ascites. When more central parts, like the thoracic duct, the cisterna chyli, or their major tributaries are affected; a fluid rich in triglycerides, featuring the presence of chylomicrons, known as chyle, accumulates, leading to conditions such as chylous ascites (chyloperitoneum), chyloretroperitoneum, chylothorax, or chylorrhea [1].

The occurrence of these leaks significantly heightens morbidity and mortality rates, primarily attributed to malnutrition resulting from protein and fat loss. Moreover, complications extend to lymphocytopenia, immunosuppression, and, in certain instances, respiratory distress. Additionally, all forms of lymphatic leaks detrimentally impact wound healing, consequently lengthening post-operative recovery periods, hospital stays, and diminishing the quality of patient care and lives [2,3].

With contemporary oncological abdominal and cardiothoracic interventions becoming more aggressive, coupled with prolonged survival rates for cancer patients, the incidence of post-operative lymphatic and lympho-chylous leaks is on the rise [4]. Consequently, the timely diagnosis and treatment of these complications have become paramount.

While the existing literature provides valuable insights, there currently exist no standardized treatment guidelines. Recommendations for the management of these post-operative leaks advocate a step-up approach, commencing with conservative measures (CMs) before considering surgical intervention [5]. Conservative strategies encompass dietary control (emphasizing high-protein, low-fat, and medium-chain triglyceride diets), fasting, total parenteral nutrition, along with the administration of drugs (such as somatostatin and octreotide analogs and diuretics), as well as the application of pressure dressings and drainage. Somatostatin and octreotide are used to diminish lymph flow and gastrointestinal secretions, while diuretics help manage fluid overload. However, these measures have been shown to take from several weeks to up to two months to alleviate symptoms [1].

In cases where conservative approaches prove ineffective, surgical revision becomes imperative. Nevertheless, most surgeons remain reluctant to attempt surgical revision and the clipping of lymphatic ducts, given the inherent difficulty of the intraoperative identification of these structures. Therefore, the need for alternative treatment algorithms is significant.

In 1921, the radiologists Jean-Athanase Sicard and Jacques Forestier serendipitously discovered Lipiodol, an iodinated poppy seed oil, for its contrast-enhancing properties in X-ray imaging. Thirty years later, Kinmonth introduced its application in visualizing the lymphatic system through lymphangiography [6]. Presently, Lipiodol lymphangiography (LL) is a valuable tool for detecting various types of lymphatic leaks, boasting a detection rate estimated to be between 64 and 78% [7]. Beyond its diagnostic role in identifying lymphatic leaks, recent reports suggest that LL may also harbor therapeutic potential. The underlying mechanism, however, remains incompletely elucidated. One hypothesis is that the viscous properties of Lipiodol or the inflammatory response induced at the disruption point may lead to the obstruction of the leakage site. Consequently, Lipiodol holds the promise of serving as both a diagnostic and therapeutic tool.

The purpose of this paper is to evaluate the therapeutic effect of LL on post-operative lymphatic leaks by performing a systematic review of the literature. This review is underlined by a case series of five patients who underwent LL in our surgical department.

## 2. Materials and Methods

### 2.1. Systematic Review of the Literature

#### 2.1.1. Selection Criteria

The systematic review of the literature was performed in accordance with the Preferred Reporting Items for Systematic Reviews and Meta-Analyses (PRISMA) guidelines [8]. The literature search was performed until July 2022 in the PubMed (Medline), Web of science, and Central (Cochrane Central Register of Controlled Trials) databases using the medical subject heading (MeSH) terms Lipiodol and lymphangiography [9]. The exact search terms were ((Lipiodol) OR (Ethiodized Oil)) AND ((Lymphangiography) OR (Lymphography)). The last search was performed on the 31st of July 2023, yielding 480 articles. To identify relevant articles, a PICOS framework was used for comprehensive and bias-free searches (Table 1). The search strategy details can be found in the Supplementary Materials (Table S1).

After the exclusion of duplicates, the titles, and abstracts of all the remaining articles were screened for eligibility by the main author. The electronic search was then supplemented by searching through the references of each individual article included. The full-text evaluation of the potentially relevant articles was then performed independently by two authors. Disagreements between the reviewers were resolved by consensus.

#### 2.1.2. Data Extraction

The primary data extraction was performed by the main author. The finally included studies were sorted into 2 groups; Group 1 included the retrospective case reviews of 5 or more patients and Group 2 included case series (<5 patients) and case reports. This division into two groups was decided upon to diminish selection bias.

The data were extracted and collected into 2 tables (Group 1/Group 2). The variables for which data were sought were: study characteristic (main author, year of publication, and title), number of patients, surgical procedure and initial diagnosis, lymphatic leak presentation, initial leak management, indication for LL, interval to LL, technical aspects of LL (access for LL, contrast quantity and injection speed, imaging post LL), outcomes (technical and clinical success), leakage volume/day before and after LL, timeframe to clinical success, complications, and bailout procedures. The retrieved data were then reviewed by all of the authors, and disagreements were resolved by consensus.

#### 2.1.3. Bias/Quality Assessment

To evaluate the validity of the included studies, a quality assessment was conducted for each study by two independent authors using the method proposed by Murad et al. [10]. A summary of the quality assessment is presented in a similar manner to that suggested by Higgins et al. in the *Cochrane Handbook for Systematic Reviews of Interventions* [11]. Disagreements were resolved by consensus.

#### 2.1.4. Statistical Analysis

Percentages, means, and medians were calculated as appropriate. Statistical analyses were performed in Microsoft Excel^®^ 2016.

### 2.2. Retrospective Case Series

To complete our literary review, we present five patients who underwent LL for post-operative chylous leaks in our surgical department.

We retrospectively analyzed all adult patients presenting lymphatic leaks in our department, who underwent LL for diagnostic and therapeutic purposes between September 2018 and December 2020. The utilized data included the patients’ demographics (age, gender), history and comorbidities, lab results, treatments, imaging studies (lymphangiography and computer tomography/X-ray follow-up), and surgical protocols. All patients had signed our institution’s general consent form for the use of data for research purposes. The study protocol was approved by the local ethical committee (BASEC ID 2021-02526; CER-VD, Swiss ethics Switzerland).

Our primary endpoint was to evaluate the efficacy of LL in diagnosing lymphatic leaks and to assess the timeframe in which any therapeutic effect was observed.

## 3. Results

### 3.1. Systematic Review of the Literature

#### 3.1.1. Study Selection

**Figure 1:**
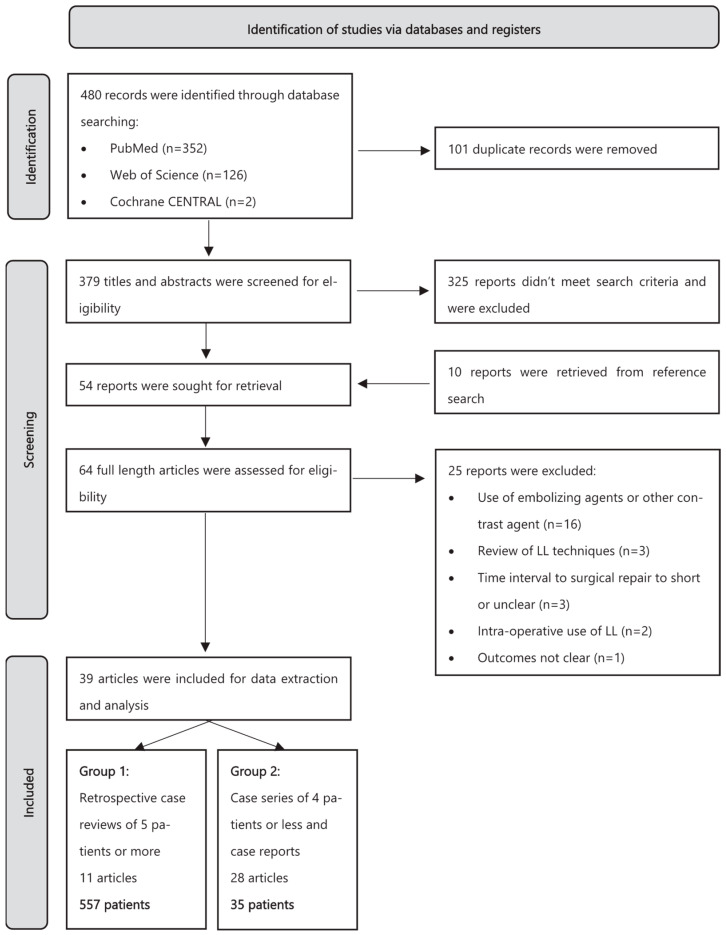
Flowchart of study selection according to PRISMA [8].

A total of 480 articles were identified by searching the above-mentioned databases. After removing 101 duplicates, 379 remained for abstract evaluation. In total, 325 articles were excluded as they did not meet our inclusion criteria. The remaining 54 reports, as well as 10 reports identified through reference lists, were assessed for eligibility. Sixteen papers were excluded because LL was coupled with other percutaneous interventions (e.g., coiling or the use of glue) and data for LL alone were not available; three articles were excluded because they were interventional technique reviews; three articles were excluded because the time to surgical ligation was less than 48 h or was unclear; two papers were excluded as LL was used in the pre-operative setting; and one paper was excluded as clinical and technical success were not specified. A summary of the study selection process is provided in Figure 1. Ultimately, 39 articles were included in the systematic review. All reviewers were in agreement, and no consensus process was required.

These 39 articles included 11 retrospective case analyses, 3 case series of four patients or fewer, and 25 individual case reports [12,13,14,15,16,17,18,19,20,21,22,23,24,25,26,27,28,29,30,31,32,33,34,35,36,37,38,39,40,41,42,43,44,45,46,47,48,49,50]. No prospective studies or randomized controlled trials could be found.

#### 3.1.2. Bias/Quality Assessment

**Figure 2:**
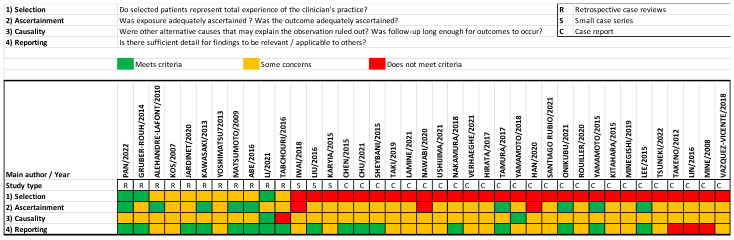
Quality assessment using Murad et al.’s proposed method of all included papers [10].

The quality assessment of the included articles using the method proposed by Murad et al. [10] is summarized in Figure 2. As all of the studies were observational, mainly case series and case reports, reporting bias was present, and the level of evidence limited.

On an important note, the case report published by Rouiller et al. [41] in 2020 (which included an author of this paper), is also included in our paper as patient C. Relevant information extracted from that case report can be found in the Supplementary Materials (Tables S3–S6).

#### 3.1.3. Extracted Data

This sections details the papers of Group 1. Due to the low level of evidence of all papers included in Group 2 (case reports and small case series), more information on this group is available in the Section 4 and in the Supplementary Materials (Tables S3–S6).

##### Diagnosis, Surgical Approach, and Initial Leak Management

Oncological diagnosis made up 51.52% (287/557) of the cases, and lymphadenectomy (LND) was undertaken in 43.98% (245/557) of cases (Table 2). However, it is possible that some cases of LND were missed as all authors did not specify whether a lymph node resection was undertaken during the tumor resection or not.

**Figure 3:**
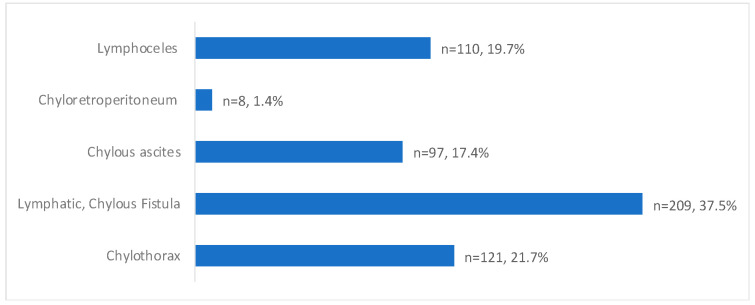
Post-operative leak presentation with percentages of total patients (Total *n* = 557).

The presentation of post-operative lymphatic leaks was 37.5% (209/557) for lymphatic or chylous fistulas, 21.7% (121/557) for chylothorax, 19.74% (110/557) for lymphoceles, 17.4% (97/557) for chylous ascites, and 1.4% (8/557) for chylo-retroperitoneum (Table 3/Figure 3).

The main component of pre-LL management consisted of nutritional management, such as total parenteral nutrition (TPN) or medium-chain triglyceride (MCT) diets, in all papers (100%; 11/11). Drainage of the lymphatic leak was undertaken in 9/11 papers, Somatostatin analogs were administered in 5/11 papers, and diuretics were used in 3/11 papers. Local management using pressure dressings was described in 3/11 papers and only 1 paper described the use of vacuum therapy.

Notably, while only 4 out of the 11 papers gave a timeframe for the use of CMs, ranging from 10 days to 3 weeks, none of the other papers specified the duration of CMs. None of these papers detailed if these CMs were continued after LL and, if yes, for how long.

##### Technical Aspects of LL

**Figure 4:**
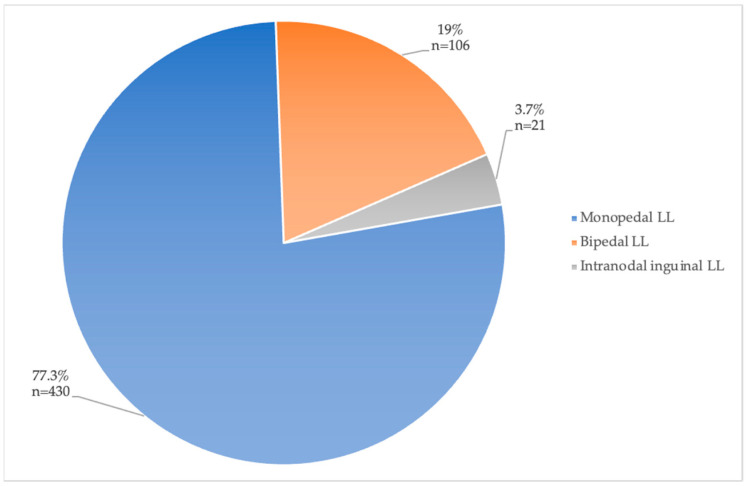
Access site for LL with percentages of total patients (total *n* = 557).

The access site for LL was either pedal (mono- or bipedal) or intranodal at an inguinal lymph node. Most papers used pedal LL (Table 4). The distribution of access sites used in all patients included in the papers of Group 1 is illustrated in Figure 4. A total of 430 patients (77.2%) underwent monopedal LL, 106 (19%) patients underwent bipedal LL, and only 21 (3.7%) underwent intranodal inguinal LL.

According to the clinical safety guidelines, the manufacturer limits the injection of Lipiodol to up to 8 mL per limb [51]. This amount was not respected in all papers.

The mean amount of Lipiodol injected was documented in seven papers, with a median value of 11.7 mL, ranging from 9.8 mL to 75 mL. Jardinet et al. used a high dosage of Lipiodol; therefore, their paper showed the highest amount of Lipiodol injected ranging from 40 mL to 140 mL with a median value of 75 mL [16].

The four remaining papers where the mean amount of injected Lipiodol was not documented, used a Lipiodol amount based on the patient’s weight with a set maximum amount (1 mL/10 kg of body weight) [14,15] or based on injection speed also with a maximum amount 1 mL/min [18,22].

For all included papers in Group 1, injection speed varied between 0.1 mL/min and 0.4 mL/min.

LL was usually guided by fluoroscopic imaging during the procedure to follow the progression of Lipiodol in the lymphatic system. Computer tomography (CT) follow-up after LL was used by Pan et al. and Gruber-Rouh et al. only if no lymphatic leaks were visualized on standard X-ray imaging. Seven out of the eleven papers used CT imaging as a standard follow-up after LL at different timepoints, which is documented in Table 4. Finally, only Alejandre-Lafont et al.. and Kos et al. used only X-ray imaging, without any mention of CT imaging.

##### Technical and Clinical Success Rates of LL

Overall, LL was technically successful in 366 out 471 patients (77.7%). Technical success was not always well defined (Table 5). When clearly defined, most papers used the definition of technical success as described by Hill et al. [52] in 2018, i.e., the uneventful cannulation of the lymphatic vessel or inguinal lymph nodes with successful injection of Lipiodol permitting opacification of the lymphatics in the area of interest. Other papers specified technical success as the successful cannulation of the lymphatic vessel or lymph node, with visualization of a Lipiodol extravasation.

Clinical success, defined as a substantially reduced post-procedure lymph volume drainage, therefor not requiring any other bailout procedures, was 62.6% (295/471 patients). The clinical success of all technically successful LLs was 80.6% (295/366 patients).

The mean amount of lymphatic leakage per day before LL varied greatly between papers and was only specified in 8/11 papers. The median value of these eight papers was 977.5 mL/day. The mean minimum amount documented among included papers was 300.4 mL/day [13], and the highest mean amount documented was 1852 mL/day [16]. However, when looking at the ranges of leakage output for each paper, some authors described patients reaching up to 3700 mL/day [22]. Leakage volume after LL was specified in 5/11 papers, only 2 of which gave mean values [18,19].

Time-to-leak resolution after LL varied between 1 and 31 days. Again, only 7/11 papers gave mean values of the time to resolution. The median number of days to leak resolution after LL was 8 days, so a little over one week. This median was calculated after excluding the papers in which the mean time to resolution of symptoms was not precisely documented.

When LL was not clinically successful (176/471), diverse bailout procedures were undertaken such as surgical revision with thoracic duct ligation (TDL) or clipping (open or thoracoscopic), lymphocele resection or fenestration, pleurodesis, the implantation of peritoneo-venous shunts, percutaneous sclerotherapy, or even low-dose radiotherapy (RT).

Finally, of the 11 included papers in this part of the analysis (Group 1), only Gruber Rouh et al. and Jardinet et al. documented post-LL complications [13,16]. Both papers had patients showing signs of Lipiodol-related pulmonary embolisms; however, no patients showed any signs of respiratory discomfort, an asymptomatic complication considered to be Grade I according to the Clavien–Dindo classification [53].

##### Qualitative Analysis

A summary of the relevant information extracted from all papers included in Group 1 is reported in the Supplementary Materials (Table S2).

### 3.2. Case Series

Five patients (one female and four male) underwent diagnostic and therapeutic LL for post-operative chylous leaks in our surgical department between September 2018 and December 2020. Table 6 summarizes the extracted data of these five patients.

#### 3.2.1. Pre-Procedure Assessment

The presence of a milky fluid in the post-operative drainage raised the clinical suspicion of lymphatic leaks in all patients. Incomplete drainage or collection was confirmed by CT scan in all patients.

The general treatment protocol was identical in all patients, with the exception of patient B. The first step in the management of the leaks consisted in local drainage; for our five patients, (post-operative) drainage was already in place. The second step of management consisted in a “nothing-by-mouth” diet and total parenteral nutrition.

LL was the third step of management, the time interval, which was non-consistent in all patients, ranged from 6 to 16 days.

#### 3.2.2. LL Interventional Technique

##### Ultrasound-Guided Intranodal Lymphangiography

Intranodal lymphangiography was performed in all patients with a high dose of ethiodized oil (Lipiodol^®^ Ultra Fluide 4.8 g/10 mL, Guerbet, Villepinte, France). The procedure consisted of an ultrasound-guided bilateral puncture of an inguinal lymph node with placement of the tip of a 23–25 G needle (BD Vacutainer^®^ 23 G or 25 G, Becton, Dickinson and Company, Franklin Lakes, NJ, USA) at the junction between the cortex and the hilum. As inguinal lymph nodes are usually small, the pubic and peripubic regions were shaved 24 h prior to the procedure in some patients to induce a mild nodal inflammatory reaction and lymph node expansion. The correct position of the needle tip was confirmed with the injection of the sulfur hexafluoride sonographic contrast (SonoVue^®^ 8 µL/mL, Bracco, Milan, Italy) and the needles were fixed with an adhesive patch. The injection of Lipiodol was performed slowly by hand under fluoroscopic control at an approximate rate of 0.5 mL/min. The fluoroscopic control was also necessary to follow the progression of Lipiodol in the lymphatic system and to verify the absence of extravasation. Approximatively 10 mL of Lipiodol was applied to each side. Dedicated Lipiodol-resistant syringes (Qitexio^®^, Guerbet, Villepinte, France) were used given the inherent risk of degradation with conventional syringes.

No complications or technical difficulties were encountered during the procedure, and no complications were observed after the intervention.

##### Post-Lymphangiography Unenhanced CT

As Lipiodol started to opacify the aortic lymph nodes, an unenhanced CT scan was performed. Half of the patients needed a second CT examination 3 to 12 h after the first scan due to the non-opacification of the target lymphatic structures. The objectives of the CT scan were (1) to evaluate the progression of Lipiodol, (2) identify the cisterna Chyli to plan an eventual subsequent direct transabdominal catheterization and embolization, and (3) eventually identify the source of the leakage.

##### Clinical and Technical Success of LL

As described by Hill et al. in 2018, we defined the technical success of lymphangiography as an uneventful cannulation of the lymphatic vessel or inguinal lymph nodes with successful injection of Lipiodol permitting opacification of the lymphatics in the area of interest. We defined clinical success as the post-procedure lymph volume being significantly reduced, therefore not requiring any other bailout procedure [52].

#### 3.2.3. Post-LL Follow-Up

All patients were monitored post-procedure in the surgical unit. For patients presenting chylothorax, saturation rates, and oxygen needs were monitored. For patients presenting chylous ascites, abdominal discomfort and signs of abdominal distention were monitored. We also followed the daily output of the post-operative drainages, quantified over 24 h.

Clinical improvement of lymphatic leaks was determined as a reduction in drainage output. As described above, clinical success was defined as a significantly reduced drainage volume, below 50 cc/day, allowing for drain removal, and therefore not requiring any other bailout procedures. In all patients, oral nutrition was re-introduced before drainage removal.

The long-term clinical follow-up of a minimum of 12 months was undertaken. No cases of lymphatic leak re-occurrence were documented.

#### 3.2.4. Case Presentation

##### Patient A—Bilateral Chylothorax Post-Transhiatal Esophagectomy

This patient was diagnosed with adenocarcinoma of the lower esophagus, leading to transhiatal esophagectomy with gastric tube creation and jejunostomy tube placement after initial neoadjuvant radio-chemotherapy.

On post-operative day (POD) 4, the presence of a milky fluid in the bilateral chest drains was noted, with an initial drain output of over 1000 mL/d, fluid analysis proved the effusion was chylous. TPN was initiated immediately; and the patient underwent LL 48 h later.

**Figure 5:**
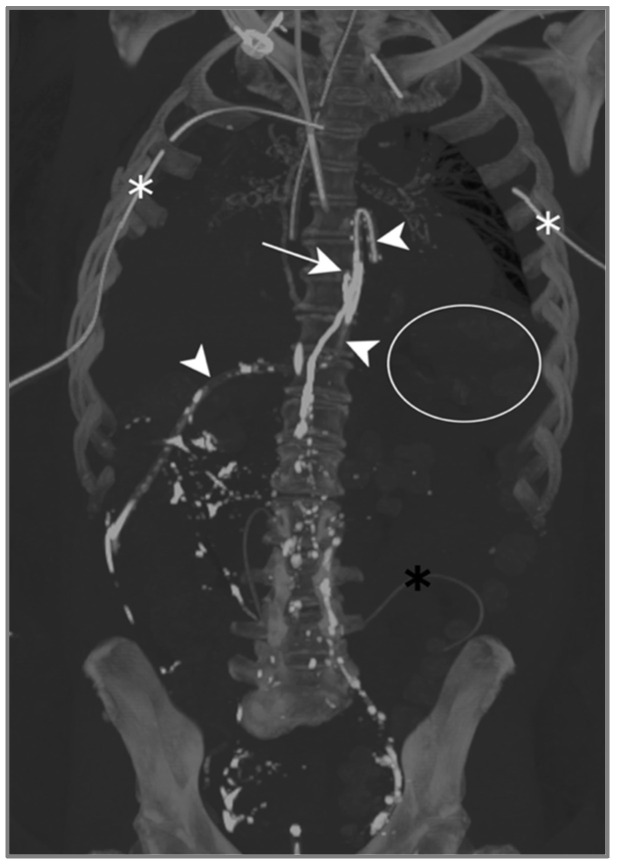
Patient A. The maximal-intensity projection of the CT scan reconstructed in the coronal plane. The Lipiodol injection shows opacification of the left inguinal and prevertebral lymphatic chains up to the level of the inferior vertebral plateau of D7 (white arrow). The drainage of the contrast agent through the catheter along the oesophagojejunal anastomosis is highlighted (white arrowheads). The subtle soft attenuation in the projection of the left lung field (white circle) corresponds to the accumulation of contrast in the pleural space due to the lymphatic rupture. The bilateral pleural catheter (white asterisks) and peritoneal drain (black asterisk) are identified.

The contrast injected in the inguinal region showed ascension into the thoracic duct up to the level of the inferior vertebral plateau of D7, as well as the leakage and accumulation of Lipiodol in both pleural spaces: Lipiodol also accumulated in the drain coming into contact with the esophago-jejunal anastomosis (Figure 5).

One day after lymphangiography, the drain output had diminished by half, and reduced to less than 100 mL/d as of day 2. Per-mouth nutrition was begun progressively, with no recurrence of chylous effusion, allowing for drain removal on day 13.

Lymphangiography was therefore successful in treating this bilateral post-operative chylothorax.

##### Patient B—Right Chylothorax Post Distal Esophagectomy

This patient was diagnosed with adenocarcinoma of the lower esophagus. After neoadjuvant radio-chemotherapy, he underwent distal esophagectomy with direct gastro-esophageal anastomosis and jejunostomy tube placement.

On POD 4, the right chest drain fluid analysis was compatible with a chylothorax, with an initial drain output of over 1000 mL/d.

**Figure 6:**
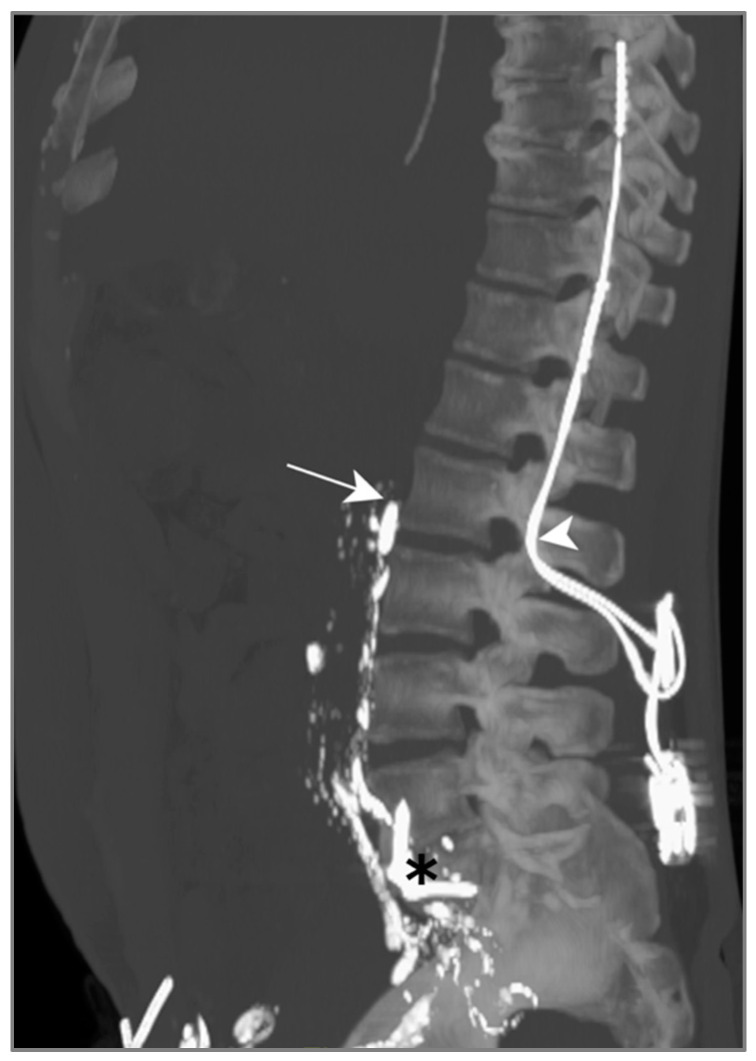
Patient B. The maximal-intensity projection of the CT scan reconstructed in the sagittal plane. The progression of Lipiodol in the prevertebral lymphatic chains is demonstrated up to the level of the 2nd lumbar vertebra (white arrow). The location of a spinal neurostimulator (white arrowhead) and anterior lumbar arthrodesis material (black asterisk), both anteriorly placed, are highlighted.

LL was attempted to visualize and potentially seal the leak, but the contrast agent did not show any progression above L2 on successive CT scans (Figure 6). We suspect this was due to a previous orthopedic intervention on the spine, inducing post-operative lymphatic tissue remodeling.

Due to an electrolyte imbalance and difficult venous access, TPN was only initiated after LL. This CM however enabled a slow drain output reduction 6 days after nutrition had been begun; no other bailout procedures were necessary.

In this case, lymphangiography was unsuccessful in treating this post-operative chylothorax, due to lymphatic duct disruption post orthopedic surgery.

##### Patient C—Right Chylothorax After Open Right Lower Lobectomy

This patient was diagnosed with lung carcinoma of the right lower lobe. After chemo- and immunotherapy, he underwent an open right sided lower lobectomy with a bronchus pericardial patch and radical mediastinal lymphadenectomy.

**Figure 7:**
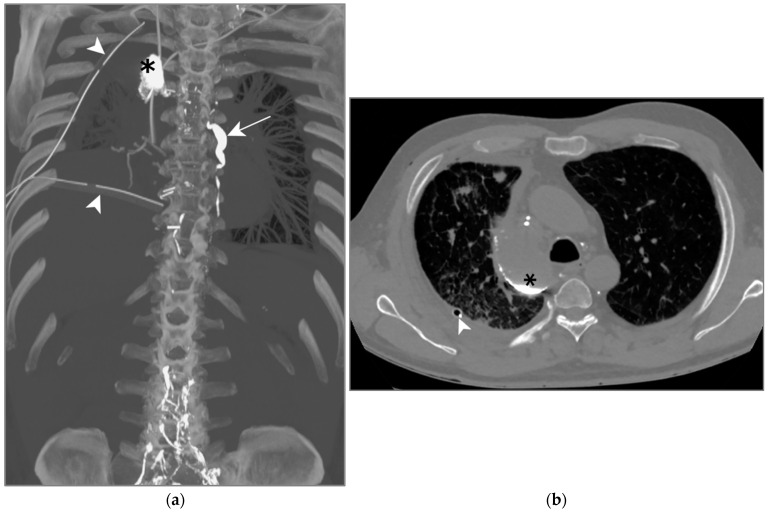
Patient C. The maximal-intensity projection of the CT scan reconstructed in the coronal plane (**a**) and the axial CT plane through the upper pulmonary lobes (**b**). (**a**,**b**) Accumulation of hyperattenuating Lipiodol in an encapsulated collection in the apical portion of the right pleural space (black asterisks). The ductus thoracicus is dilatated (white arrow). Two right pleural catheters are shown (white arrowheads).

A right sided, high-output chylothorax (>900 mL/d) was diagnosed on post-operative day 1, TPN was immediately begun. Ten days after TPN, drain output was still high leading to surgical revision. A re-thoracotomy with ductus thoracicus ligation was attempted, but unsuccessful. An LL was then attempted to identify the location of the leak. Lymphography was technically successful, showing images of extravasation from the mediastinal lymph nodes in the right pleural space (Figure 7a,b). The reason for the persistence of chylothorax was a bilateral thoracic duct.

This intervention enabled drain output to be reduced by half but did not succeed in sealing the leak. The problem was finally solved by a VATS left sided thoracic duct ligation. This case was previously described by Rouiller et al. in 2020 [41].

##### Patient D—Right Chylothorax Post Thoracoscopic Lobectomy

This patient was diagnosed with lung carcinoma of the right medium lobe and underwent curative thoracoscopic lobectomy of the right medium lobe.

**Figure 8:**
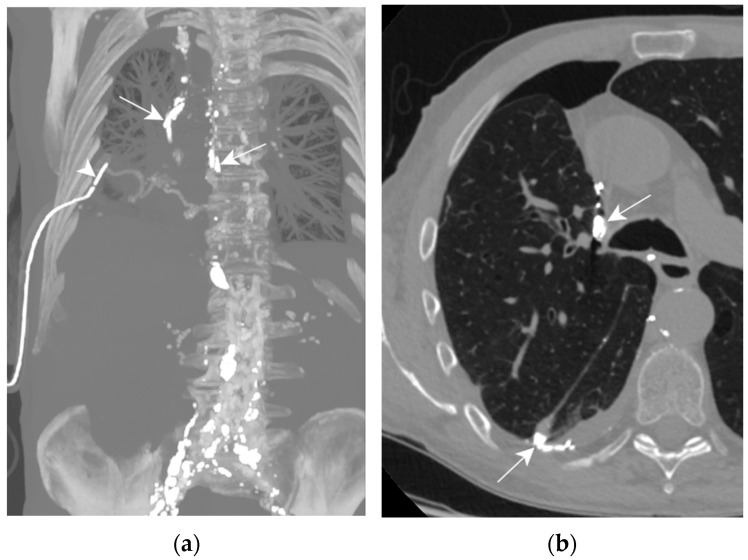
Patient D. The axial CT plane through the upper pulmonary lobes with magnification on the right hemithorax (**b**); the maximal-intensity projection of the CT scan reconstructed in the coronal plane (**a**). (**a**,**b**) The presence of Lipiodol in a right pleural effusion along the mediastinum and in its declive portion (white arrows). Thoracic drainage with a pleural catheter (white arrowhead) is shown.

On post-operative day 4, a high-output right chylothorax was diagnosed (>1500 mL/d). TPN was immediately begun and maintained for 10 days; high-volume leakage persisted (Figure 8a,b). On day 10, the patient underwent an LL which showed extravasation of contrast agent into the right pleural cavity. Three days after the LL, the drain output had diminished to less than 100 mL/d, allowing drain removal on day 5. The LL was successful in treating this one-sided post-operative chylothorax.

##### Patient E—Chylous Ascites After Pancreaticoduodenectomy

This patient was diagnosed with peri-ampullar duodenal adenocarcinoma and underwent pancreaticoduodenectomy (the Whipple procedure) and nutritional jejunostomy tube placement.

**Figure 9:**
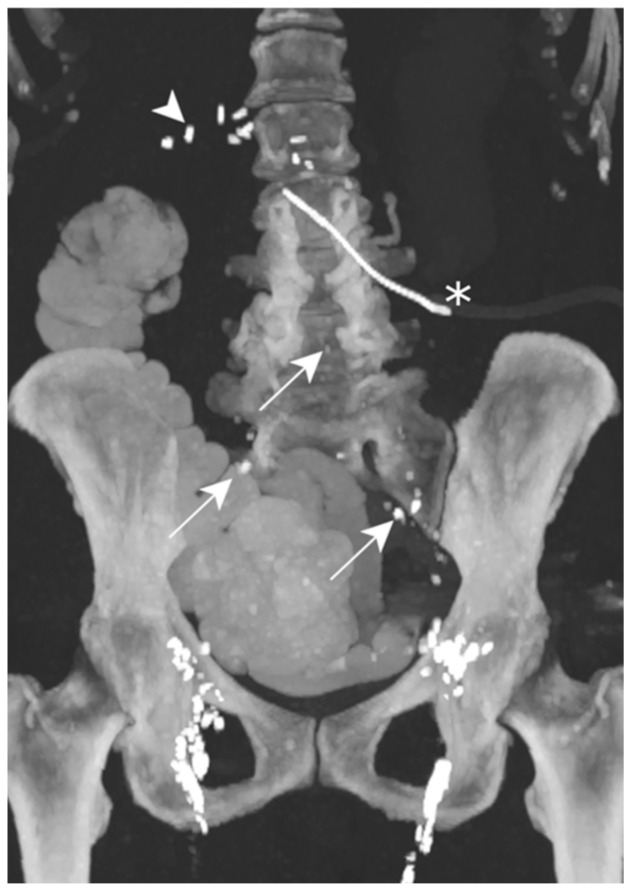
Patient E. The maximal-intensity projection of the CT scan reconstructed in the coronal plane after a transnodal Lipiodol injection, showing contrast agent in the external and common iliac as well as retroperitoneal (white arrows) lymphatic chains. No leakage of contrast agent is visualized. The presence of radiopaque surgical material is evident in the projection of the upper right quadrant, and is related to the cephalic duodenopancreatectomy (white arrowhead). The peritoneal drainage catheter (white asterisk) is identified.

On POD 7, the abdominal post-surgical drain showed a milky substance of up to 750 mL/d. TPN was begun and, 48 h later, she underwent an LL. The images did not show the precise lymphatic leakage origin, but contrast agent was visible in the drainage catheter (Figure 9).

Less than 72 h after the lymphangiography, the drain output diminished to less than 60 mL/d, allowing for drain removal on POD 14.

The lymphangiography was successful in treating this post-operative chylous ascites.

#### 3.2.5. Technical and Clinical Success—Case Series

A total of five patients underwent LL for post-operative leaks. Four patients developed chylothorax, one of which was bilateral. One patient presented chylous ascites.

##### Technical Success:

LL was technically successful in all but one patient (an 80% technical success rate). For the patient in which LL was not technically successful, there was no progression of the Lipiodol above L2 due to a previous orthopedic procedure, probably leading to an interruption of the lymphatic vessels at this level.

Thankfully, this patient showed a decrease in chylous leakage one week after the initiation of TPN, allowing for drain removal without further intervention.

##### Clinical Success:

All four patients where LL was technically successful showed a decrease in leak output. In three out of the four patients, LL sufficiently decreased the chylous leak, allowing drainage removal without further procedure (a success rate of 75%).

In one case, LL allowed for some drain output decrease, but not this was not sufficient for drain removal. This was certainly due to the presence of a bilateral thoracic duct. The chylothorax was finally solved by thoracoscopic left sided thoracic duct ligation.

No patients had further post-intervention complications.

## 4. Discussion

CMs play a crucial role in the management of chylous leaks, offering initial strategies to alleviate symptoms and promote healing without relying on invasive interventions. The main conservative approach involves dietary modifications, such as a low-fat diet. Temporary fasting may also be recommended, allowing the digestive system to rest, and reducing the demand on the lymphatic system. Furthermore, the incorporation of medium-chain triglycerides (MCTs) into the diet may be explored, as MCTs are absorbed directly into the bloodstream and can serve as an alternative energy source, bypassing the lymphatic system’s involvement in fat absorption.

In addition, other CMs are helpful such as compression therapy, limb elevation, or drain placement.

The use of somatostatin or its analog Octreotide, administered subcutaneously or intravenously, is primarily considered when CMs prove insufficient and more invasive interventions are not immediately warranted. Somatostatin has been explored in the management of chylous leaks due to its ability to inhibit the release of various gastrointestinal hormones (gastrin, cholecystokinin, and secretin); it suppresses the production and secretion of chyles, including those responsible for stimulating lymphatic flow. Somatostatin works by suppressing the production and secretion of chyle, the milky lymphatic fluid containing dietary fats. It achieves this by inhibiting the release of hormones like gastrin, cholecystokinin, and secretin, which play a role in stimulating the digestive process and subsequent lymphatic flow. By reducing the volume of chyle produced, somatostatin helps alleviate the pressure on the lymphatic system, contributing to the resolution of chylous leaks.

The reviewed literature does not precisely clarify which measures should be started, neither their application timeframe nor the length of their application after LL.

If, after implementing all these measures, the leaks persist, surgical revision is considered, but it can be challenging due to the intricacies of the lymphatic system and the early post-operative setting. However, LL emerges as a potentially promising alternative.

LL for the detection of diverse lymphatic leaks is a well-established and valuable procedure. Its primary goal is to visualize lymphatics and/or lymph nodes through fluoroscopic guidance, achieved by injecting Lipiodol intra-lymphatically in the inguinal or pedal region. This visualization not only aids in accurately identifying the location and extent of lymphatic leaks but also proves beneficial in strategically planning surgical revisions for any post-operative occurrences of lymphatic leaks.

Studies have revealed that LL holds promise not only in the detection but also in the treatment of lymphatic leaks. The proposed mechanism for LL’s therapeutic efficacy involves the accumulation of Lipiodol, introduced during lymphography, at the site of leakage outside the lymphatic vessel. This accumulation triggers a localized inflammatory response in the surrounding soft tissue, ultimately leading to the obstruction of the leakage point. Additionally, the compression exerted by the cystic mass formed from the leaked chyle may contribute to the obstruction at the point of lymphatic vessel leakage [19].

Our systematic review of the literature is an exhaustive aggregation of the current relevant information on the use of LL in a post-operative setting. The 11 retrospective studies show the current technical aspects of LL and the technical and clinical success rates for the treatment of various kinds of lymphatic leaks.

The technical aspects of LL were comparable between papers as this is a well-established procedure. Access sites were either pedal or intranodal in the inguinal region. Of the 11 papers included in Group 1, only one paper used inguinal injection of Lipiodol [16]. In Group 2, on the other hand, most papers used an intranodal inguinal access site.

Lipiodol was used as a contrast agent in all of the papers, as this was set in our inclusion criteria, and the injection speed was similar in all papers (0.1 mL/min). The only variation in the technical aspects could be seen in the quantity of contrast agent chosen to be injected. Some papers chose to follow the manufacturers guidelines, others used higher doses of Lipiodol in the hope of better visualizing the leaks and obstructing the leakage point.

Imaging was mainly undertaken using X-ray/fluoroscopy during lymphography. Conventional tomography can also be used during but also after LL to demonstrate Lipiodol progression in the lymphatic system. This enables visualization of the signs of leakage—direct leakage, i.e., extravasation, or indirect leakage, i.e., contrast agent pooling. The timepoint or window of imaging within which to visualize the leaks is the filling phase (performed during contrast agent propagation into the central lymphatic system), and additionally the nodal phase (performed in multiple articles 24 h after contrast material injection if X-ray imaging in the filling phase remains unclear) [54].

Finally, to discuss the technical and clinical success, we divided the identified relevant papers into two groups based on their level of evidence, whilst hopefully diminishing the selection bias. As paper’s included in Group 2 were mainly case reports and three small case series of three to four patients, the clinical success rates were close to 100%, as the author’s chose to showcase their positive experiences of using LL in patients presenting with post-operative lymphatic leaks. No firm conclusion can therefore be drawn from these 28 articles; however, we included the extracted data in the Supplementary Materials (Tables S3–S6) of this paper to gain information and knowledge on the current uses of LL.

Information extracted from the papers included in Group 1 suggests that LL is effective in up to 80.6% of cases for the treatment of lymphatic leaks. Meaning that, thanks to LL, a noticeable reduction in lymphatic output was observed, thereby allowing the removal of the drainage and no need for further, more invasive, surgical procedures.

Factors associated with treatment failure were a high leak output (>500 mL/day) and Lipiodol extravasation on post-LL imaging [12]. Some authors believe that if active extravasation can be seen on post LL imaging, surgical ligation should be undertaken.

Yoshimatsu et al. observed the correlation between radiological finding and the success of LL. If the leak appeared nodular on the post-LL CT, clinical success was 100%, if, however, the leak appeared beaded, clinical success was only 40%. A “nodular” appearance might suggest that the leak presents as discrete, rounded, well-defined structures on imaging, which may indicate a more localized leak at the leakage point. A “beaded” appearance could imply that the leak appears as a series of connected, elongated structures resembling beads on a string, suggesting a more complex or extensive pattern of leakage, without stagnation at the leakage point. The speculation behind this finding is that the nodular appearance reflected the local stagnation of iodized oil, which led to a regional inflammatory reaction more quickly [18]. This finding could help decision making for the management of the leak, not only based on the drainage output but also on the CT findings after lymphography.

**Figure 10:**
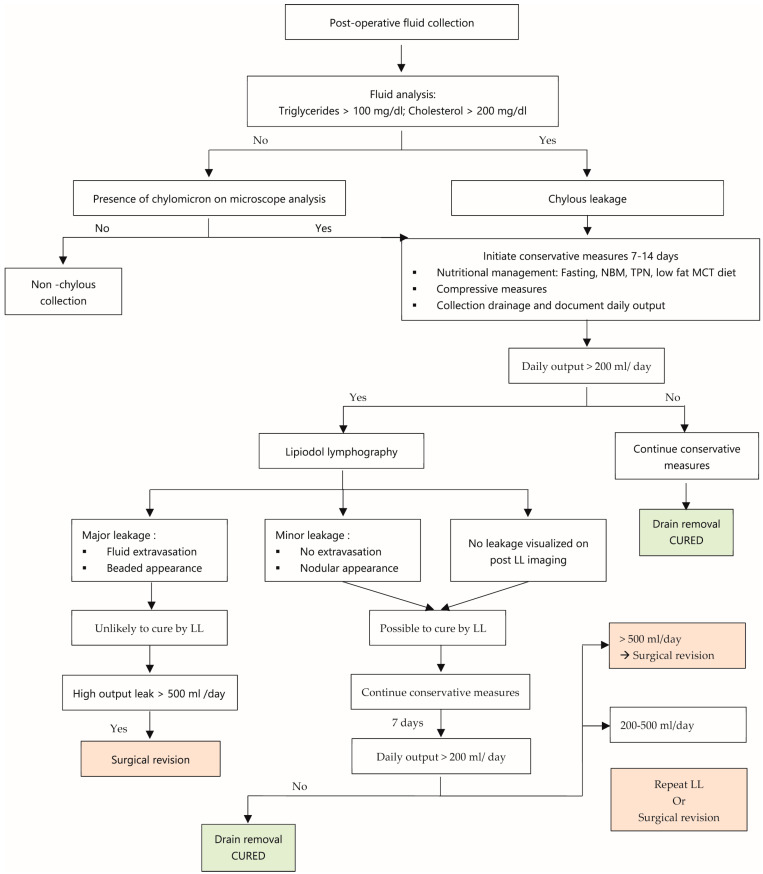
Post-operative lymphatic leak management algorithm.

The analyzed data did not reveal clear guidelines for the management of these leaks. Based on this systematic review, we present a post-operative lymphatic leak management algorithm (Figure 10). In order to further evaluate the efficacy of CMs and LL for the treatment of lymphatic leaks with this algorithm, prospective randomized studies will be necessary.

LL is a safe procedure if the contraindications are respected. Current strict contraindications to lymphography include pulmonary insufficiency and a right-to-left cardiac shunt, as these predispose the patients to respiratory complication (risk of pulmonary embolism) or cerebral embolism. Also, LL should not be performed in cases of lymphedema, as this can worsen the condition by further obstructing the lymphatic system [12,15,28]. In this review, only two papers in Group 1 reported Lipiodol pulmonary embolisms, patients thankfully were asymptomatic. In Group 2, Sheybani et al. reported one case of cerebral embolism following LL and Taki el al. reported one case of severe ARDS [28,29].

Even though the indication for LL was always a post-operative lymphatic leak, the clinical background leading to surgery, the causal surgery, and the presentation of the leaks varied greatly among the included papers. Also, the volume of the leaks, and the previously attempted CMs were inconsistent. The timeframe between the diagnosis of a lymphatic leak and LL was unclear and the duration/continuation of CMs was not clearly specified.

In our systematic literature review on LL, several limitations should be acknowledged. Firstly, the studies included in the review varied considerably in terms of patient group sizes, surgical interventions, and clinical indications, spanning a publication range from 2007 to 2022. This heterogeneity may limit the comparability of results and generalizability of our findings. Additionally, while a bias analysis was conducted, the overall quality of the papers was low due to their predominantly descriptive nature, making it difficult to draw strong conclusions from the existing literature. Another significant limitation is that many studies did not clearly indicate whether CMs were continued following lymphography, which may have influenced outcomes. As a result, we present our management algorithm as a preliminary template that requires further validation. Future randomized controlled trials will be essential to substantiate and refine this algorithm to ensure its clinical applicability and reliability.

## 5. Conclusions

LL is a safe procedure with a high technical success rate. As well as enabling direct visualization of the post-operative leakage point, in 62.6% of cases, LL is also a curative measure. Further randomized control trials are necessary to determine optimal CM, timing of these measures and the timing and indications for LL.

## Figures and Tables

**Table 1:** Scope of the literature review in PICOS framework.

PICOS	Inclusion	Exclusion
Population	Adult patients > 18 years of age Presenting post-operative lymphatic leakage	Non-post-operative lymphatic leaks LL performed in pediatric cases
Intervention	LL for diagnostic or therapeutic purposes	LL using alternative contrast agent and the use of glue/coils/other percutaneous procedures for embolization
Comparison	Conservative treatment measures of lymphatic leaks	N/A
Outcome	Therapeutic and clinical success of LL	No report of therapeutic and clinical success of LL
Study design	Clinical studies and case series	Animal and cadaveric studies Reviews, congress abstracts, letters

Abbreviations: Lipiodol lymphography (LL); not applicable (N/A).

**Table 2:** Study characteristics, surgical indication, and surgical intervention with percentages—Group 1.

Author/Year	Title	N	Surgical Indication	Surgical Intervention
Pan/ 2022 [12]	Treatment of postoperative lymphatic leakage applying transpedal lymphangiography—experience in 355 consecutive patients	258 (355)	Terminal renal insufficiency (20.8%) Abdominal carcinoma/sarcoma (16.6%) PAOD (10.1%) Malignant melanoma (6.5%) Aortic aneurysm/dissection (5.9%) Pelvic carcinoma/sarcoma (5.4%) Vulvar squamous cell carcinoma (4.8%) Esophagus carcinoma (4.5%) Coronary heart disease (3.4%) Valvular heart disease (2.0%) Other benign diseases (11.3%) Other malignant tumors (8.7%)	Exlap with tumor resection (26.8%) Kidney transplantation (18.9%) Transfemoral catheterization (14.9%) Cutaneous/s.c. tumor resection (14.9%) Peripheral artery bypass (6.8%) Thoracic-abdominal esophagus resection (5.4%) Vulvectomy (3.9%) S.c. hematomectomy with vascular repair (2.5%) Explorative thoracotomy (0.8%) Radical thyroid tumor resection (0.8%) Artificial femoral head replacement (0.8%) Others (3.4%)
Gruber-Rouh /2014 [13]	Direct lymphangiography as treatment option of lymphatic leakage: Indications, outcomes, and role in patient’s management	71	Cutaneous Melanoma (52.1%) Merkel Cell Carcinoma (4.2%) Ovarian cancer (2.8%) Other (40.9%)	Inguinal LND (52.1%) Pelvic LND (2.8%) Renal transplantation (14%) Radical prostatectomy (9.8%) Cystectomy (2.8%) Splenectomy (1.4%) Esophagectomy (9.8%) Gastrectomy (7%)
Alejandre-Lafont/ 2010 [14]	Effectiveness of therapeutic lymphography on lymphatic leakage	33	Bronchial carcinoma (10%) Aneurysmal disease (6%)) Osteochondrosis (2%) Coronary heart disease (4%) Liver cirrhosis (8%) Lymphangiomyomatosis (2%) Morbus Hodgkin (2%) Non-Hodgkin lymphoma (8%) Esophageal carcinoma (14%) Sarcoidosis (2%) Pancreatitis (2%) Appendicitis (2%) Nephrotic syndrome (8%) Endometrium carcinoma (2%) Testicular carcinoma (4%) Bladder carcinoma (4%) Melanoma (2%) Penile carcinoma (2%) Prostate carcinoma (2%)	Pneumectomy + LND (10%) Cystectomy (6%) Spondylodesis (2%) Cardiac bypass (4%) Esophagectomy + LND (14%) Duodeno-pancreatectomy (2%) Appendectomy (2%) Kidney transplantation (8%) Hysterectomy + LND (2%) Cystectomy (4%) LND (6%)
Kos/ 2007 [15]	Lymphangiography: forgotten tool or rising star in the diagnosis and therapy of postoperative lymphatic vessel leakage	22	Bronchial carcinoma (9%) Renal cell carcinoma (4.5%) Polycystic kidney disease (4.5%) Esophageal carcinoma (9%) Melanoma (18%) M. Hodgkin (4.5%) Vulval carcinoma (4.5%) Penile carcinoma (4.5%) Myasthenia gravis (4.5%) Aneurysmal disease (9%) Embryonic carcinoma (4.5%) Prostate carcinoma (4.5%) Coronary heart disease (4.5%) PAOD (4.5%) Gastric carcinoma (4.5%) Thigh seroma (4.5%)	Pneumectomy (9%) Nephrectomy (9%) Esophagectomy (9% LND (18%) Thymectomy (18%) Endoaneurysmoraphy (4.5%) Semicastration + retroperitoneal LND (9%) Prostatectomy (4.5%) Vascular bypass (9%) Partial gastrectomy (4.5%) Soft-tissue resection (4.5%)
Jardinet/ 2020 [16]	Intranodal lymphangiography with high-dose ethiodized oil shows efficient results in patients with refractory, high-output postsurgical chylothorax: a retrospective study	18	N.S.	Esophagectomy (72%) Lobectomy (10%) Explorative thoracotomy (4.5%) Pericardiectomy (4.5%) Nissen fundoplication (4.5% Pericardiectomy (4.5%)
Kawasaki/ 2013 [17]	Therapeutic effectiveness of diagnostic lymphangiography for refractory postoperative chylothorax and chylous ascites: correlation with radiologic findings and preceding medical treatment	14	Gastric cancer (14.2%) Esophageal carcinoma (42.8%) Esophageal melanoma (7.1%) Aortic valve stenosis (7.1%) Dissected aneurysm (7.1%) Thoracoabdominal aortic aneurysm (14.2%) Ruptured thoracic aortic aneurysm (7.1%)	Partial esophagectomy (28.5%) Total esophagectomy (14.2%) Thoracic/thoracoabdominal aortic replacement (28.5%) Aortic valve replacement with mitral valvuloplasty (7.1%) Distal gastrectomy (14.2%) Subtotal esophagectomy (7.1%)
Yoshimatsu/ 2013 [18]	Prediction of therapeutic effectiveness according to CT findings after therapeutic lymphangiography for lymphatic leakage	14	Esophageal cancer (42.8%) Gastric cancer (7.1%) Rectal cancer (7.1%) Annulo-aortic ectasia (14.2%) Scoliosis (7.1%) Colon cancer (7.1%) Testicular cancer (14.2%)	Esophagectomy (42.8%) Gastrectomy (7.1%) Proctectomy (7.1%) Aortic root replacement (7.1%) Surgery for scoliosis (7.1%) Colectomy (7.1%) Retroperitoneal (7.1%) LND (14.2%)
Matsumoto/ 2009 [19]	The effectiveness of lymphangiography as a treatment method for various chyle leakages	9	Esophageal cancer (44.5%) Breast cancer (11.1%) Ovarian cancer (11.1%) Testicular cancer (11.1%) Annuloaortic ectasia (11.1%)	Esophagectomy (44.5%) Axillary LND (11.1%) Pelvic LND (11.1%) Retroperitoneal LND (11.1%) Aortic root replacement (11.1%)
Abe/ 2016 [20]	Therapeutic strategy for chylous leakage after esophagectomy: focusing on lymphangiography using Lipiodol	9	Esophageal cancer (100%)	Subtotal esophagectomy and LND via right thoracotomy and laparotomy (100%)
Li/ 2021 [21]	Preliminary exploration of transpedal lymphangiography with high-dose ethiodized oil application in the treatment of postoperative chylothorax	7	Oesophageal carcinoma (28.5%) Non-small-cell lung carcinoma (71.5%)	Pulmonary resection + mediastinal LND (57.1%) Sublobar resection + mediastinal LND (14.3%) Esophagectomy + mediastinal LND (28.6%)
Tabchouri/ 2016 [22]	Chylous ascites management after pancreatic surgery	5	Cancer as primary diagnosis	Pancreatic surgery (100%)

Abbreviations: peripheral artery occlusive disease (PAOD); explorative laparotomy (Exlap); subcutaneous (S.c.); lymphadenectomy/lymph node dissection (LND); not specified (N.S.).

**Table 3:** Lymphatic leak presentation with percentages, initial management, and timeframe—Group 1.

Author/Year	Lymphatic Leak Presentation	Leak management Before LL	Timeframe of Conservative Measures
Pan/ 2022 [12]	Cervical chylous fistula (1.6%) Chylothorax (10.1%) Chylous ascites (21.3%) Chylo-retroperitoneum (2.7%) Pelvic lymphocele (20.5%) Lymphocutaneous fistula (43.8%)	Nutrition management Somatostatin analogs Drainage Wound vacuum therapy	More than 2 weeks
Gruber-Rouh /2014 [13]	Lymphatic fistula (52.1%) Lymphocele (15.49%) Chylothorax (18.3%) Chylous ascites (14%)	Nutrition management: TPN Drainage Pressure dressing	More than 3 weeks
Alejandre-Lafont/ 2010 [14]	Chylothorax (60%) Lymphocele (18%) Chylous ascites (6%) Chylothorax + chylous ascites (8%) Chylous ascites + lymphocele (8%)	Nutrition management: TPN or MCT Diet Iterative drainage/paracentesis Compression bandages Diuretics Sclerotherapy	No timeframe
Kos/ 2007 [15]	Chylothorax (36.4%) Chylous ascites (4.5%) Lymphatic fistula (41%) Lymphocele (13.6%) Chylothorax + chylous ascites (4.5%)	Nutrition management: TPN or MCT Diet Compression Drainage	No timeframe/in addition to LL
Jardinet/ 2020 [16]	Bilateral chylothorax (39%) Unilateral chylothorax (61%)	Nutrition management: TPN or MCT Diet Attempt at TDL	No timeframe/in addition to LL
Kawasaki/ 2013 [17]	Chylothorax (78.5%) Chylous ascites (14.2%) Chylothorax + chylous ascites (7.14%)	Nutrition management: NBM, TPN Drainage Octreotide (300 μg/day) Attempt at TDL/pleurodesis	No timeframe
Yoshimatsu/ 2013 [18]	Chylothorax (50%) Chylous ascites (35.7%) Lymphocele (7.1%) lymphatic fistula (7.1%)	Nutrition management: TPN or MCT Diet Drainage	No timeframe
Matsumoto/ 2009 [19]	Chylothorax (55.5%) Chylous ascites (22.2%) Lymphatic fistula (22.2%)	Nutrition management: TPN or MCT Diet Drainage	No timeframe
Abe/ 2016 [20]	Chylothorax (100%)	Nutrition management: TPN or MCT Diet Drainage Octreotide acetate	No timeframe
Li/ 2021 [21]	Chylothorax (100%)	Nutrition management: TPN Somatostatin	More than 2 weeks
Tabchouri/ 2016 [22]	Chylous ascites (100%)	Nutrition management: NBM, TPN, MCT Diet Drainage Somatostatin for 3 days	For 10–14 days

Abbreviations: Lipiodol lymphangiography (LL); total parenteral nutrition (TPN); medium-chain triglyceride (MCT); thoracic duct ligation (TDL); nothing by mouth (NBM).

**Table 4:** Technical aspects of LL—Group 1.

Author/Year	Access Site for LL	Lipiodol Quantity and Injection Speed	Imaging Post LL
Pan/ 2022 [12]	Monopedal (88.7%) Bipedal (11.3%)	10 mL (7–14) At 0.1–0.5 mL/min for an hour	X-ray: 0–1 h, 1–3 h, and 4–6 h If indeterminate Lipiodol extravasation: CT If Lipiodol extravasation is still not marked, CT at 24 h
Gruber-Rouh /2014 [13]	Monopedal (66.1%) lymphatic fistula or lymphocele Bipedal (33.8%) chylothorax or chylous ascites	11.7 mL (4–20) At 6–8 mL/h	X-ray overview of the feet and legs If the lymphatic leakage was not visible, about 24 h after the procedure unenhanced CT was performed
Alejandre-Lafont/ 2010 [14]	Monopedal (92%) Bipedal (8%)	1 mL/10 kg, max 20 mL At 5–10 mL/h	X-ray shortly after starting the injection and again after finishing the injection (about 2 h after start of injection)
Kos/ 2007 [15]	Monopedal (91%) Bipedal (9%)	1 mL/10 kg per foot, max 14 mL At 4–7 mL/h	X-ray during filling phase plus late-phase after about 24 h
Jardinet/ 2020 [16]	Inguinal intranodal (100%)	75 mL (40–140) Speed not specified	Radiographic guidance Cone-beam CT
Kawasaki/ 2013 [17]	Bipedal (100%)	16 mL total (8/foot) Injection pressure 1.5–2.0 kg/cm^2^	CT and X-ray from the pelvis to chest just after LL
Yoshimatsu/ 2013 [18]	Bipedal (100%)	8 mL/foot not t exceeding a volume of 12 mL At 0.1 mL/min	CT and X-ray
Matsumoto/ 2009 [19]	Monopedal (55.5%) Bipedal (45.5%)	9.8 mL (6–12) At 0.1 mL/min	Abdominal + chest X-ray 2 h after LL Native CT 5–28 h after LL
Abe/ 2016 [20]	Monopedal (66.6%) Intranodal (33.3%)	10 mL At 0.1 mL/min	Abdominal X-ray 1–2 h after LL Chest and abdominal X-ray + CT 2–6 h after LL
Li/ 2021 [21]	Monopedal (100%)	27.6 mL (21.2–30) At 0.4 mL/min	Fluoroscopy every 2–5 min until opacification of the left jugular vein angle CT immediate and at 24 h
Tabchouri/ 2016 [22]	Bipedal (100%)	12 mL maximum over 30–45 min	An abdominal and pelvic CT scan 120 min after LL

Abbreviations: Lipiodol lymphangiography (LL); computer tomography (CT).

**Table 5:** Technical and clinical success rates of LL as documented in papers—Group 1.

Author/Year	Indication for LL	Time to LL	Technical Success	Clinical Success	Mean Daily Leakage Before LL	Mean Daily Leakage After LL	Mean Leakage Timeframe After LL	Bailout Procedures
Pan/ 2022 [12]	Failed CM for >2 weeks	26 days (16–49 days)	169/258 (65.5%)	159/258 (61.6%) 159/169 (94%)	500 mL (300–1100 mL)	N.S.	5 days (2–7 days)	Surgical revision, percutaneous sclerotherapy, low-dose RT, and continuous CM
Gruber-Rouh /2014 [13]	Unlikely to be cured by CM for >3 weeks	N.S. (>21 days)	64/71 (90.1%)	45/71 (63.3%) 45/64 (70.3%)	300.4 mL (10–1000 mL)	N.S. <200 mL (*n* = 33) >200 mL (*n* = 31)	N.S. (10 days–4 weeks)	Surgical revision
Alejandre-Lafont/ 2010 [14]	Failed CM/surgical intervention too risky No timeframe	24 days (3–117 days)	43/49 (87.7%)	27/49 (55.1%) 27/43 (63%)	N.S. (500–2500 mL)	N.S.	N.S. (1–14 days)	TDL or resection of the lymphocele
Kos/ 2007 [15]	N.S.	N.S. (5–154 days)	20/22 (90%)	11/22 (50%) 11/20) (55%)	N.S. (200–3000 mL)	N.S.	N.S. (Maximum 3 weeks)	Lymphocele fenestration, surgical clipping
Jardinet/ 2020 [16]	Failed CM/Post TDL No timeframe	28 days (4–104 days)	17/18 (94%)	15/18 (83%) 11/14 (78%) *	1852 mL (525–3760 mL)	N.S.	12 days (1–25 days)	TDL
Kawasaki/ 2013 [17]	Failed CM No timeframe	13.5 days (3–62 days)	14/14 (100%)	9/14 (64.3%)	950 mL (300–3000 mL)	N.S. 2–100 mL (*n* = 5) 100–200 mL (*n* = 4) >700 mL (*n* = 5)	8 days (3–29 days)	TDL or pleurodesis
Yoshimatsu/ 2013 [18]	Unlikely to be cured by CM No timeframe	N.S	14/14 (100%)	8/14(57%)	1005 mL (150–3000 mL)	806 mL (50–3000 mL)	N.S.	Pleurosclerosis Implantation of a peritoneovenous shunt
Matsumoto/ 2009 [19]	Unlikely to be cured by CM No timeframe	N.S. (10–42 days)	9/9 (100%)	8/9(89%)	533 mL (150–1500 mL)	256 mL (50–1500)	17 days (4–31 days)	TDL
Abe/ 2016 [20]	Failed CM No timeframe	15 days (6–23 days)	9/9 (100%)	2/9(22.2%)	N.S. <500 mL (*n* = 1) 500–1000 mL (*n* = 2) >1000 mL (*n* = 6)	N.S. <500 mL (*n* = 5) 500–1000 mL (*n* = 1) >1000 mL (*n* = 3)	13 days (4–21 days)	Chemical pleurodesis or TDL/clipping
Li/ 2021 [21]	Failed CM for >2 weeks	20 days (15–31 days)	7/7 (100%)	6/7(86%)	1500 mL (1100–2000 mL)	N.S.	7 days (4–13 days)	Percutaneous afferent lymphatic vessel sclerotherapy
Tabchouri/ 2016 [22]	N.S.	30 days (15–55 days)	N.S.	5/5(100%)	1700 mL (600–3700 mL)	N.S.	7.6 days (2–21 days)	None

Abbreviations: conservative measures (CM); thoracic duct ligation (TDL); not specified (N.S.); radiotherapy (RT); * 5 patients had 2 LL.

**Table 6:** Case overview—Retrospective case series.

Age/Gender (Unique I.D)	Surgical Indication/Intervention	Lymphatic Leak Presentation	Management Before LL	Technical Aspects of LL	Technical Success	Clinical Success	Mean Daily Leakage Before LL	Mean Daily Leakage After LL	Bailout Procedures	Length of Stay
67 F (A)	Esophageal carcinoma/Esophagectomy	Bilateral Chylothorax	TPN	15 mL POD6	Yes	Yes	1080 mL	Day 1: 550 mL Day 4: 50 mL	-	25 days
46 M (B)	Esophageal carcinoma/Esophagectomy	Unilateral Chylothorax	N.S.	10 mL POD6	No *	No	1000 mL	1000 mL No change	Back to CM—TPN	20 days
62 M (C)	Bronchial carcinoma/Lobectomy	Unilateral Chylothorax	TPN TDL	10 mL POD16	Yes	No	900 mL	Day 3: 500 mL **	TDL	35 days
68 M (D)	Bronchial carcinoma/Lobectomy	Unilateral Chylothorax	TPN	20 mL POD14	Yes	Yes	1700 mL	Day 1: 60 ml	-	21 days
67 M (E)	Duodenal carcinoma/Lobectomy	Chylous Ascites	TPN	15 mL POD8	Yes	Yes	750 mL	Day 1: 95 mL Day 3: 60 mL	-	15 days

Abbreviations: female (F); male (M); not specified (N.S.); total parenteral nutrition (TPN), thoracic duct ligation (TDL), post-operative day (POD). * no cranial progression of Lipiodol above L2; ** insufficient reduction.

## Data Availability

Data for the case series are available upon reasonable request from the corresponding author. The full dataset from the systematic review can be found in the Supplementary Materials provided with this article.

## Supplementary Materials

The following supporting information can be downloaded at: https://www.mdpi.com/article/10.3390/jcm13216432/s1, Table S1. Systematic review of the literature, search strategy; Table S2. Qualitative analysis of papers included in Group 1; Table S3. Study characteristics, surgical indication and surgical intervention with percentages—Group 2; Table S4. Lymphatic leak presentation with percentages, initial management, and timeframe—Group 2; Table S5. Technical aspects of LL—Group 2; Table S6. Technical and clinical success rates of LL as documented in papers—Group 2.
