# Supplementary material for: Ultrasound-Guided Intranodal Lipiodol Lymphangiography for the Assessment and Treatment of Chylous Leaks: A Retrospective Case Series from a Single Center in Switzerland and a Systematic Review of the Literature
Source: J Clin Med. 2024 Oct 27;13(21):6432. doi: 10.3390/jcm13216432 (PMC11547011; doi:10.3390/jcm13216432)
Supplement: Supplementary file 1 [file jcm-13-06432-s001.zip › jcm-3258608-supplementary.pdf]

Journal of
*Clinical Medicine*

MDPI

# Supplementary materials

**Table S1.** Systematic review of the literature, search strategy

| Database | Search Terms |
|---|---|
| PubMed/Medline | ((Lipiodol) OR (Ethiodized Oil)) AND ((Lymphangiography) OR (Lymphography)) |
| Web of science | ((Lipiodol) OR (Ethiodized Oil)) AND ((Lymphangiography) OR (Lymphography)) |
| Cochrane / central | ((Lipiodol) OR (Ethiodized Oil)) AND ((Lymphangiography) OR (Lymphography)) |

**Table S2.** Qualitative Analysis of papers included in Group 1

| | **Various types of lymphatic leaks** |
|---|---|
| **Pan/ 2022** | The authors enrolled the largest cohort of patients of all papers included in this analysis. A total of 355 patients underwent transpedal lymphography for a confirmed post-operative lymphatic leakage. Most of these patients underwent malignant tumor resection with simultaneous lymphadenectomy. Lymphography took place between 17-54 days after causal surgery, indication for it was failed conservative treatments for more than 2 weeks (nutrition management, somatostatin analogs, drainage, wound vacuum therapy). Of the 355 patients, LL was technically unsuccessful for 41 patients; only 258/355 underwent LL alone, and the remaining 56 patients underwent simultaneous LL and invasive treatment (surgical revision or sclerotherapy). The 258 patients undergoing LL alone are therefore the only ones of interest for our analysis. Technical success was 100% as the 41 cases where LL failed, were excluded from the analysis. 159/258 (61.1%) had a clinically successful LL, after a median of 5 days (IQR: 2–7 days). A univariate logistic regression analysis revealed that a post-operative lymphatic leak output of > 500 ml/d and Lipiodol extravasation on post LL imaging, were significantly correlated with treatment failure of LL (p = 0.025 and 0.014, respectively). |
| **Gruber-Rouh/2014** | The authors published their experience of LL in 71 patients presenting post operative lymphatic leaks refractory to conservative therapy. LL was technically successful in 64/71 patients (90.1%), and clinically successful 45/71 patients 63.3% (70.3% out of technically successful group). The leak persisted between 10 days and 4 weeks after LL. The remaining 19 patients underwent surgical intervention for complete occlusion; the timeframe of the surgical bailout procedures was however not mentioned, neither was the surgical indication. The volume of lymphatic drainage per day was a significant predictor of the therapeutic success rate (p = 0.002). If drainage was less than 200 ml per day, LL was successful in 96.8% of cases, above 200 ml /day, the success rate fell to 58.1%. |
| **Alejandre-Lafont/ 2010** | 49 patients underwent LL after primary diagnosis of lymphatic leakage. Treatment with LL was undertaken after failed conservative treatment (TPN or MCT Diet, iterative drainage / paracentesis, compression bandages, diuretics), the timeframe of which was unclear (3-117 days). Technical success was 87.7%, clinical success was 55.1% of all performed LL, 63% of all successful LL.<br>The likelihood ratio test showed that neither the age of the patient nor the cause of lymph duct damage, or the period up to the performance of the therapeutic LL had a significant influence on the therapeutical success. However, the volume of lymphatic drainage per day was a here again a significant factor. Patients with a drainage volume of less than 500 mL/d offered a two-fold higher success rate (70%) compared to patients with more than 500 mL/d of lymphatic drainage (35%). |
| **Kos/ 2007** | The authores intended to diagnose and treat 22 patients with post operative lymphatic leaks with LL. Technical success was 90%, clinical success was 50%. It is important to note, that in this paper, conservative treatment was clearly continued after LL (compression, drainage, and total parenteral nutrition) for 3 weeks in most of the cases. 5 patients underwent early surgical revision, the reason of which is unclear. In the subgroup analysis chylothorax (75%), chylaskos (100%), chylothorax plus chylaskos (100%), and lymphatic fistula (85.7%) showed very high rates of therapeutic success without the need for further surgical intervention. None of the lymphocele cases however were treated sufficiently with conservative management and LL alone. |
| **Kawasaki/ 2013** | 14 patients presenting lymphatic leaks after gastrointestinal surgery (9/14) or cardiovascular surgery (5/14). The time between leak diagnosis and LL was between 3-62 days (median 13.5 days), conservative measures consisted in nutrition management (NBM, TPN), drainage, octreotide (300 μg/day) or TDL attempt (1/14) pleurodesis (1/14). Bipedal LL was technically successful in all patients. The leak output before treatment ranged from 300 to 3000 mL/d (median, 950 mL/d). LL was clinically successful in 9/14 patients (64.3%) by 3–29 days (median, 8 days). The 5 remaining patients maintained an output of more than 700 ml /day after follow-up of 8–29 days (median, 15 days), resulting in surgical ligation (4 patients) or pleurodesis (1 patient). |
| **Yoshimatsu/ 2013** | 14 patients were included, all underwent bipedal lymphangiography for post operative leaks (chylothorax 50%, chylous ascites 35.7%, lymphocele 7.1% and lymphatic fistula 7.1%). The mean time to LL was not specified, conservative measure consisted in nutrition management (TPN or MCT Diet) and drainage. Technical success was 100%, with no related complications. LL was clinically successful in 8/14 patients (57%). In 3 of the 6 patients with clinical failure, additional treatments such as pleuro-sclerosis (n = 2) and implantation of a peritoneovenous shunt (n = 1) were performed. The bailout procedure of the remaining 3 cases was not specified. |
| **Matsumoto/ 2009** | The authors evaluated the use of pedal LL for post operative chylothorax (55.5%), chylous ascites (22.2%) and lymphatic fistula (22.2%) in a total of 9 patients. Technical success was 100% with no related complications. In 7 of the 9 patients (78%), the daily amount of drainage decreased the day after lymphangiography. On an important note, the authors of this paper made it clear that conservative measures were maintained after LL, the timeframe of which was not mentioned. In one of the remaining two patients, the daily amount of drainage remained the same on the day after lymphangiography but conservative measures were continued because the daily amount of chyle leakage was relatively small (70ml / day). Therefor 8/9 patients needed no surgical reintervention and lymphatic |

| | |
|---|---|
| | leakage stopped after lymphangiography (89% success rate). The remaining patient underwent surgical occlusion, because the daily amount of drainage remained at more than 1500 ml for 7 days after lymphangiography. |
| **Chylothorax** | |
| **Jardinet/ 2020** | The authors published their experience of using high dose ethiodized oil based inguinal LL specifically for post-operative chylothoraxes (7 patients with bilateral chylothorax, 11 patients with unilateral chylothorax). They also limited their study population to patients suffering from leaks of more 500 cc / day. The mean timeframe to inguinal LL was 28 days (4-104 days), during which conservative measures were used (medium-chain triglyceride–enriched diet and total parenteral nutrition). 11 of the 18 patients underwent thoracic duct ligation before LL, this was successful in 4 cases. The remaining 7 showed a persistent leak after revision surgery (3 patients showed incomplete ligation of the TD, 4 patients showed complete ligation with collateral lymph vessels supplying the leak. LL was attempted in all 18 patients, even after thoracic-duct ligation attempt. Technical success was achieved in all but one patient (17/18; 94%). The only case of technical failure was caused by retroperitoneal lympho-venous shunting. Clinical success was evaluated by the authors at 83% (15/18 patients), however 4 of those patients had already undergone successful TDL, if excluded the clinical success would be 78%.Another important note is that 5 patients underwent a second LL because of increasing chest tube output after initiation of a medium-chain triglyceride–enriched diet. Three of these five patients (60%) had complete resolution after the second INL. The two remaining patients had clinical failure. |
| **Abe/ 2016** | Analysis of the effect of LL for chylothorax after subtotal esophagectomy and lymphadenectomy via right thoracotomy and laparotomy in 9 patients with oesophageal cancer. The median time until LL was 15 days (range of 6–23 days), during which nutrition management, drainage and octreotide acetate was given. Technical success of pedal LL (6/9) and inguinal LL (3/9) was 100%, showing chylous leakage from the main trunk of the thoracic duct (main trunk type) in 2 patients, while 6 had leakage from the branches of the thoracic duct (branch type). Clinical success however was only 22.2% (2/9 patients), the 7 remaining needed chemical pleurodesis (3/7) or video assisted clipping (4/7). It is important to note however that 6/7 patients presented a high-volume leak of more than 500 cc/day. |
| **Li/ 2021** | Paper evaluating post-operative unilateral chylothorax, was Li et al's published in 2021, summarizing their experience in 7 patients. All patients underwent surgical tumor resection and lymphadenectomy for esophageal carcinoma (2/7) or non-small cell lung carcinoma (5/7). Monopedal LL was performed at a median of 20 days (range: 15–31 days) after surgery. All patients presented a high-volume leak, the median daily chyle output before TL was 1,500 ml/day (range: 1,100–2,000 ml/day). The technical success of TL was 100% (7/7). After LL, 6 patients experienced a progressive decrease of the daily chyle output. The median time for removing the thoracic drainage after TL was 7 days (range: 4–13 days), clinical success was measured at 86% (6/7). The remaining patient underwent percutaneous afferent lymphatic vessel sclerotherapy on the 8th day after LL and was cured. |
| **Chylous ascites** | |
| **Tabchouri/ 2016** | 15 patients presenting chylous ascites after pancreatic surgery. 10 patients received conservative measures alone, 5 patients underwent additional bipedal LL. Fifteen patients (100 %) received TPN and 13 (86.7 %) received low-fat MCT rich enteral feeding. Ten patients received both treatments simultaneously at some point. All patients received somatostatin analog for at least 3 days. If conservative treatments showed no improvement, bipedal LL was attempted as of day 10-14.No patients required any additional surgical measures to treat their CA. Bipedal LL resulted in CA resolution in all 5 patients (100 % clinical success). |

**Table S3.** Study characteristics, surgical indication and surgical intervention with percentages – Group 2

| Author / Year | Title | N | Surgical Indication | Surgical Intervention |
|---|---|---|---|---|
| Iwai/ 2018 | Experience of Lymphangiography as a Therapeutic Tool for Lymphatic Leakage After Kidney Transplantation | 4 | Diabetic nephropathy (75%)<br>Nephrosclerosis (25%) | Living kidney transplantation |
| Liu/ 2016 | Ultrasound-guided intranodal lipiodol lymphangiography from the groin is useful for assessment and treatment of post-esophagectomy chylothorax in three cases | 3 | Esophageal squamous cell carcinoma (100%) | Radical esophagectomy with LND (33%)<br>Robot-assisted minimally invasive esophagectomy with three-field LND (33%)<br>Thoracoscopic esophagectomy with LND (33%) |
| Kariya/ 2015 | Repeated intranodal lymphangiography for the treatment of lymphatic leakage | 3 | Esophageal cancer (33%)<br>Rectal cancer + intestinal perforation (33%)<br>Malignant Lymphoma (33%) | Esophagectomy, cervical transverse incision (33%)<br>Rectal and ileocecal resection with a peritoneal drainage tube placement (33%)<br>Right inguinal LND (33%) |
| Chen/ 2015 | Therapeutic role of ultrasound-guided intranodal lymphangiography in refractory cervical chylous leakage after neck dissection: Report of a case and review of the literature | 1 | Squamous cell carcinoma of the tongue | Right partial glossectomy with radical neck dissection, then right parotidectomy |
| Chu/ 2021 | Management of intractable post-adrenalectomy chylous ascites with microsurgical intra-abdominal lymphaticovenous anastomosis: A case report and literature review | 1 | Metastasis from lung cancer | Left laparoscopic adrenalectomy |
| Sheybani/ 2015 | Cerebral Embolization of Ethiodized Oil following Intranodal Lymphangiography | 1 | Sclerosing mesenteritis | Exploratory laparoscopy with peritoneal washings and intraoperative omental and peritoneal biopsies |
| Taki/2019 | A case of acute respiratory distress syndrome due to lymphography with Lipiodol for chylothorax after esophagectomy | 1 | Lower esophageal cancer | Video-assisted thoracoscopic esophagectomy with three-field lymph node dissection and hand-assisted laparoscopic surgery |
| Lamine/ 2021 | Lipiodol lymphangiography as a treatment for refractory postoperative chylothorax: a case report | 1 | Primary lung adenocarcinoma | Right upper sleeve lobectomy combined with a S6 segmentectomy and radical mediastinal lymph node dissection |

| | | | | |
|---|---|---|---|---|
| Nawabi / 2020 | Lymphangiography with lipiodol as a diagnostic and therapeutic approach for Chyle Leak ascites following Simultaneous Pancreas-Kidney Transplant | 1 | Diabetic end stage kidney desease | Simultaneous Pancreas-Kidney Transplant |
| Ushijima/ 2021 | Successful treatment of intractable chylous ascites after laparoscopic low anterior resection using lymphangiography and embolization with lipiodol: A case report | 1 | Rectal cancer | Laparoscopic low anterior resection with D3 lymph node dissection |
| Nakamura /2018 | Ultrasound-guided intranodal lymphangiography with lipiodol for treatment of chylous ascites following surgery for ovarian cancer: A case report | 1 | Ovarian Carcinoma | Total hysterectomy, bilateral salpingo-oophorectomy, omentectomy, and pelvic and para-aortic LND |
| Verhaeghe/ 2021 | Postoperative lymphocele causing obturator nerve entrapment, treated with percutaneous drainage and intranodal poppyseed oil (Lipiodol)-based lymphangiography | 1 | Prostate adenocarcinoma | Robot-assisted laparoscopic prostatectomy and bilateral pelvic LND |
| Hirata/ 2017 | CT-Guided Intranodal Lymphangiography for Postoperative Chylous Ascites | 1 | Extrahepatic bile duct cancer | Percutaneous transhepatic biliary drainage, pylorus-preserving pancreaticoduodenectomy |
| Tamura/ 2017 | Cervical chylous leakage following esophagectomy that was successfully treated by intranodal lipiodol lymphangiography: a case report | 1 | Middle thoracic esophageal cancer | Radical esophagectomy with 3-field lymph node dissection |
| Yamamoto / 2018 | Chylothorax after hepatectomy: a case report | 1 | Cholangio tubular adenocarcinoma | Right hepatectomy, caudate lobectomy, extrahepatic bile duct resection and LND |
| Han / 2020 | Refractory Lymphatic Ascites After Radical Prostatectomy | 1 | Prostate adenocarcinoma | Robotic transperitoneal radical prostatectomy with standard pelvic LND |
| Santiago Rubio / 2021 | Giant Idiopathic Lymphocele 18 Years After Kidney Transplantation, Treated Using Lymphatic Embolization With Lipiodol: Report of a Rare Case | 1 | End-stage kidney disease | Kidney transplant |
| Onikubu / 2021 | Computed tomography-guided lymphangiography from the para-aortic lymph node: a useful approach for chylothorax after esophagectomy | 1 | Middle thoracic esophageal squamous cell carcinoma | Esophagectomy |
| Rouiller/2020 | Bilateral thoracic duct ligation for persisting postoperative chylothorax | 1 | Lung IIIA squamous cell carcinoma | Open lower bilobectomy with radical mediastinal LND |
| Yamamoto / 2015 | Chylothorax After Esophagectomy Cured by Intranodal Lymphangiography: A Case Report | 1 | Thoracic esophageal cancer | Subtotal esophagectomy and 3-field LND via right thoracotomy and laparotomy |
| Kitahara/2015 | Management of Aortic Replacement-Induced Chylothorax by Lipiodol Lymphography | 1 | Descending aortic dissected aneurysm | Descending aortic replacement via posterolateral thoracotomy. |
| Minegishi / 2019 | Massive lymphatic leakage after lung cancer surgery via median sternotomy | 1 | Non-small cell squamous Lung carcinoma | Right upper lobectomy and nodal dissection via median sternotomy |
| Lee/ 2015 | Thoracic Duct Embolization with Lipiodol for Chylothorax due to Thoracic Endovascular Aortic Repair with Debranching Procedure | 1 | Distal arch aneurysm of the aorta | Thoracic endovascular aortic repair |
| Tsuneki / 2022 | A case of aortoduodenal fistula presenting with postoperative lymphatic leakage | 1 | Intestinal erosion | Prosthetic aortic graft replacement and duodenectomy |
| Takeno /2012 | Chyluria after ligation of the thoracic duct: a rare complication after thoracoscopic-assisted esophagectomy for esophageal cancer | 1 | Advanced esophageal cancer with regional lymph node metastasis | Transthoracic excision of the esophagus assisted by thoracoscopy, with excision of the azygos vein and thoracic duct, esophagostomy and tube gastrostomy |
| Lin/ 2016 | Chylous ascites after pedicled transverse rectus abdominis myocutaneous flap harvest | 1 | Breast cancer | Right modified radical mastectomy with axillary sentinel lymph node biopsy and immediate breast reconstruction with a pedicled TRAM flap |
| Mine/ 2008 | Post-esophagectomy chylous leakage from a duplicated left-sided thoracic duct ligated successfully with left-sided video-assisted thoracoscopic surgery | 1 | Thoracic esophageal cancer | Esophagetomy via right thoracotomy with three-field LND |
| Vázquez-Vicente / 2018 | Management of a postsurgical chylous ascites following a laparoscopic retroperitoneal para-aortic lymphadenectomy | 1 | Epidermoid carcinoma of the cervix | Retroperitoneal para- aortic LND |

Abbreviations : Lymph node dissection (LND)

**Table S4.** Lymphatic leak presentation with percentages, initial management – Group 2

| Author / Year | Lymphatic leak presentation | Leak management before LL |
|---|---|---|
| Iwai/ 2018 | Lymphocele (100%) | N.S. |
| Liu/ 2016 | Chylothorax (100%) | TPN, Octreotide (2 Weeks), Unsuccessful clipping, Etilefrine hydrochloride (9 Days) |
| Kariya/ 2015 | Lymphocele (66%)<br>Chylous ascites (33%) | TPN, Intermittent drainage , Embolisation, Surgical dissection |
| Chen/ 2015 | Lymphocele | TPN, Pressure dressings, Drainage, Fibrin Glue, Sclerotherapy |
| Chu/ 2021 | Chylous ascites | Low Fat diet, TPN Somatostatine |
| Sheybani/ 2015 | Chylous ascites | Diuretic therapy |
| Taki/2019 | Chylothorax | TPN |
| Lamine/ 2021 | Chylothorax | MCT diet, TPN, Somatostatin |
| Nawabi / 2020 | Chylous ascites | High-protein, low-fat, MCT diet |

| | | |
|---|---|---|
| Ushijima/ 2021 | Chylous ascites | Nutrition management : High protein, low fat, MCT diet, Octreotide Diuretics,Drainage |
| Nakamura /2018 | Chylous ascites | Nutrition management : Fat restrictive diet , TPN , Octreotide, Drainage |
| Verhaeghe/ 2021 | Lymphocele | Drainage |
| Hirata/ 2017 | Chylous ascites | Nutrition management: MCT diet , Diuretics |
| Tamura/2017 | Cervical chylous leakage | Nutrition management: Elemental diet (Elental), Octreotide , Etilefrine |
| Yamamoto / 2018 | Chylous ascites and chylothorax | Nutrition management: TPN , Octreotide |
| Han / 2020 | Chylous ascites | N.S. |
| Santiago Rubio / 2021 | Lymphocele | Drainage |
| Onikubu /2021 | Chylothorax | Nutrition management: TPN , Octreotide |
| Rouiller/2020 | Chylothorax | Nutrition management :TPN, Open supradiaphragmatic ligation of the TD on the right side |
| Yamamoto / 2015 | Chylothorax | Nutrition mangement: TPN, Octreotide |
| Kitahara/2015 | Chylothorax | Nutrition management : Non-fat diet , TPN |
| Minegishi / 2019 | Chylothorax | Nutrition management: TPN ,Right sided thoracic ligation |
| Lee/ 2015 | Chylothorax | Nutrition management: Fat-free diet, NBM, Octreotide, Drainage |
| Tsuneki / 2022 | Chylous ascites | Nutrition management: Low fat diet , Peritoneal drainage, Diuretics |
| Takeno /2012 | Chyluria | Nutrition management : TPN |
| Lin/2016 | Chylous ascites | Nutrition management: low-fat diet, MCT diet, TPN |
| Mine/ 2008 | Mediastinal chylous leak | Nutrition management: TPN,Octreotide |
| Vázquez-Vicente / 2018 | Retroperitoneal lymphocele | Nutrition management: Low fat diet, MCT diet, TPN, Drainage, Somatostatin |

Abbreviations : Not specified (N.S.)

**Table S5.** Technical aspects of LL– Group 2

| Author / Year | Access site for LL | Lipiodol quantity and injection speed | Imaging post LL |
|---|---|---|---|
| Iwai/ 2018 | Intranodal | 8 ml at 1ml/3 min | Not specified |
| Liu/ 2016 | Intranodal | 8 ml over 66 min | CT |
| Kariya/ 2015 | Intranodal (axillary/cervical) | 5 ml /2 ml at 1 ml/3 min | Fluoroscopic guidance |
| Chen/ 2015 | Intranodal | 6 ml N.S. | Fluoroscopic guidance + CT |
| Chu/ 2021 | Intranodal | N.S. | N.S. |
| Sheybani/ 2015 | Intranodal | 25 ml N.S. | N.S. |
| Taki/2019 | Pedal | 8.5 ml at 0.1 ml/min | Videofluoroscopy /CT |
| Lamine/ 2021 | Intranodal | N.S. | N.S. |
| Nawabi / 2020 | Not specified | N.S. | N.S. |
| Ushijima/ 2021 | Intranodal | N.S. | N.S. |
| Nakamura /2018 | Intranodal | 12 ml at 6 ml/h | Fluoroscopic guidance |
| Verhaeghe/ 2021 | Intranodal | 25 ml N.S. | N.S. |
| Hirata/ 2017 | Intranodal | 5 ml N.S. | CT |
| Tamura/2017 | Intranodal | 10 ml at 1ml/5min | Fluoroscopic guidance |
| Yamamoto / 2018 | Intranodal | N.S. | CT |
| Han / 2020 | Not specified | N.S. | Fluoroscopic guidance |
| Santiago Rubio / 2021 | Intranodal | N.S. | Fluoroscopic guidance |
| Onikubu /2021 | Intranode (paraaortic) | 14 ml N.S. | CT |
| Rouiller/2020 | Intranodal | N.S. | CT |
| Yamamoto / 2015 | Intranodal | 14 ml 1 ml / 5 min | Fluoroscopic guidance |
| Kitahara/2015 | Intranodal | 15 ml N.S. | CT |
| Minegishi / 2019 | Intranodal | 7 ml N.S. | CT |
| Lee/ 2015 | Intranodal | 6-12 ml N.S. | Fluoroscopic guidance |
| Tsuneki / 2022 | Intranodal | N.S. | CT |
| Takeno /2012 | Not specified | N.S. | N.S. |
| Lin/2016 | Intranodal | N.S. | CT |

| | | | |
|---|---|---|---|
| Mine/ 2008 | Not specified | N.S. | CT |
| Vázquez-Vicente / 2018 | Intranodal | 10 ml N.S. | N.S. |

Abbreviations: Not specified (N.S.)

**Table S6.** Technical and clinical success rates of LL as documented in papers – Group 2

| Author / Year | Indication for LL | Time to LL | Technical success | Clinical success | Mean leakage volume/day before LL | Mean leakage volume/days after LL | Mean leakage timeframe after LL | Bailout procedures |
|---|---|---|---|---|---|---|---|---|
| Iwai/ 2018 | N.S. | 11-42 days | 75% | 75% | 170-360 ml | N.S. | 8-13 days | No |
| Liu/ 2016 | Failed CM | 12-93 days | 100% | 33% | 500-2000 ml | 300-1400 ml | 14-106 days | Surgical ligation |
| Kariya/ 2015 | Failed embolization/ CM | Up to 6 months | 100% | 100% | 400 ml | 20-40 ml | N.S. | Sequential LL |
| Chen/ 2015 | Failed CM | 24 days | yes | Yes | 1000 ml | 60 ml | 31 days | No |
| Chu/ 2021 | Failed CM | N.S. | yes | no | 2460 ml | No reduction | 61 days | Chylovenous bypass |
| Sheybani/ 2015 | Failed CM | N.S. | Yes | N.S. | N.S. | N.S. | N.S. | N.S. |
| Taki/2019 | Failed TPN | 9 days | Yes | Yes | 1500 ml | 200 ml | N.S. | No |
| Lamine/ 2021 | Failed CM at 3 weeks | ~ 21 Days | Yes | Yes (2 LL) | 600 ml | N.S. | N.S. | No - 2nd LL |
| Nawabi / 2020 | Failed CM | ~ 16 Days | Yes | Yes | N.S. | N.S. | N.S. | No |
| Ushijima/ 2021 | Failed CM | 93 days | Yes | Yes (2 LL) | ~ 550 ml | N.S. | N.S. | No - 2nd LL |
| Nakamura /2018 | Failed CM | 62 days | Yes | yes | 800-1000 ml | N.S. | 28 days | No |
| Ver-haeghe/ 2021 | N.S. | 28 days | Yes | Yes | 15 ml | N.S. | 2 days | No |
| Hirata/ 2017 | Failed CM | 33 days | No | Yes | 1500 ml | N.S. | 16 days | No |
| Tamura/2017 | Failed CM | 8 days | Yes | Yes | 200-300 ml | Decreased | 2 days | No |
| Yamamoto / 2018 | Recurrence after stopping CM | 62 days | Yes | Yes | 2000 ml | 500 ml | 106 days | No |
| Han / 2020 | N.S. | N.S. | Yes | Yes | 3-6L / 7-14 days | Decreased | N.S. | No |
| Santiago Rubio / 2021 | N.S. | N.S. | Yes | Yes | 800- ml | 200 ml | 7 days | No |
| Onikubu /2021 | Failed CM for 20 days | 21 days | Yes | Yes | 1500 ml | Decreased | 5 days | No |
| Rouil-ler/2020 | Failed CM and surgical measures | 20 days | Yes | N.S. | N.S. | N.S. | N.S. | TDL |
| Yamamoto / 2015 | Failed CM for 6 days | 15 days | Yes | Yes | 2000 ml | Decreased | 10 days | No |
| Kita-hara/2015 | Failed CM | 6 days ? | Yes | Yes | 300 ml | Decreased | 1 day | No |
| Minegishi / 2019 | Failed CM and surgical measures | 6 days | Yes | Yes | 2000 ml | Decreased | 14 days | No |
| Lee/ 2015 | Failed CM for 4 weeks | 46 days | Yes | Yes | 300 ml | Decreased | 3 days | No |
| Tsuneki / 2022 | Failed CM for 26 days | 26 days | Yes | Yes | N.S. | Decreased | 7 days | No |
| Takeno /2012 | N.S. | N.S. | Yes | Yes | N.S. | Decreased | 7 days | No |
| Lin/2016 | N.S. | N.S. | Yes | Yes | 70 cc/hour | Decreased | 4 days | No |
| Mine/ 2008 | Failed CM | N.S. | Yes | No | 800-1000 ml | No reduction | N.S. | TDL |
| Vázquez-Vicente / 2018 | Failed CM for 15 days | 15 days | Yes | Yes | 1000 ml | Decreased | 7 days | No |

Abbreviations: Not specified (N.S.); Conservative measures (CM); Thoracic duct ligation (TDL)
